# A Semi-Physiologically Based Pharmacokinetic Pharmacodynamic Model for Glycyrrhizin-Induced Pseudoaldosteronism and Prediction of the Dose Limit Causing Hypokalemia in a Virtual Elderly Population

**DOI:** 10.1371/journal.pone.0114049

**Published:** 2014-12-02

**Authors:** Ruijuan Xu, Xiaoquan Liu, Jin Yang

**Affiliations:** Key Laboratory of Drug Metabolism and Pharmacokinetics, China Pharmaceutical University, Nanjing, China; Cedars-Sinai Medical Center, United States of America

## Abstract

Glycyrrhizin (GL) is a widely used food additive which can cause severe pseudoaldosteronism at high doses or after a long period of consumption. The aim of the present study was to develop a physiologically based pharmacokinetic (PBPK) pharmacodynamic (PD) model for GL-induced pseudoaldosteronism to improve the safe use of GL. Since the major metabolite of GL, glycyrrhetic acid (GA), is largely responsible for pseudoaldosteronism via inhibition of the kidney enzyme 11β-hydroxysteroiddehydrogenase 2 (11β-HSD 2), a semi-PBPK model was first developed in rat to predict the systemic pharmacokinetics of and the kidney exposure to GA. A human PBPK model was then developed using parameters either from the rat model or from in vitro studies in combination with essential scaling factors. Kidney exposure to GA was further linked to an I_max_ model in the 11β-HSD 2 module of the PD model to predict the urinary excretion of cortisol and cortisone. Subsequently, activation of the mineralocorticoid receptor in the renin-angiotensin-aldosterone-electrolyte system was associated with an increased cortisol level. Experimental data for various scenarios were used to optimize and validate the model which was finally able to predict the plasma levels of angiotensin II, aldosterone, potassium and sodium. The Monte Carlo method was applied to predict the probability distribution of the individual dose limits of GL causing pseudoaldosteronism in the elderly according to the distribution of sensitivity factors using serum potassium as an indicator. The critical value of the dose limit was found to be 101 mg with a probability of 3.07%.

## Introduction

Glycyrrhizin (GL), one of the major components in licorice root, is widely used as a sweetener in food products and chewing tobacco. In addition, GL is of clinical importance as an antiinflammatory and hepatoprotective agent in the treatment of dermatitis, gastritis, hepatitis and other conditions [1]. However, several studies have reported that excessive or long-term consumption of GL can induce adverse effects such as hypertension, hypokalemia and sodium retention, all of which are symptoms of pseudoaldosteronism. In serious cases, the outcome can be death [2].

The mechanism of this pseudoaldosteronism seems to be clear [3]. Glycyrrhetic acid (GA), the main metabolite of GL, has been verified to be the major substance leading to pseudoaldosteronism. GA is a potent inhibitor of 11β-hydroxysteroiddehydrogenase 2 (11β-HSD 2), the enzyme that converts cortisol to cortisone in the kidney. This inhibition leads to an increase in cortisol levels as well as in the cortisol∶cortisone ratio in plasma and urine and, as cortisol and aldosterone have the same affinity for the mineralocorticoid receptor (MR) [4], to increased stimulation of the MR leading to increased electrolyte levels and the symptoms of pseudoaldosteronism.

The metabolism of GL depends on its route of administration [5]. GL administered by the intravenous (i.v.) route is partially hydrolyzed in the liver by lysosomal β-D-glucuronidase to 3-monoglucuronyl-glycyrrhretinic acid (3MGA). GL and 3MGA can then be excreted into the bile via canalicular transporters and finally reach the intestinal lumen. There GL and 3MGA are metabolized by the intestinal microbiota to GA, which then undergoes enterohepatic recycling. When GL is administered by the oral route (p.o.), the intestinal microbiota bring about its hydrolysis to GA which again undergoes enterohepatic recycling.

In assessing the risk of a food additive, the traditional method of measuring the acceptable daily intake, following the rule of ‘dividing by ten’, is arbitrary and provides an inaccurate estimation. It is a feasible and recommended method when the mechanism of action for the compound in question is not quite clear. However, a large number of in vivo and in vitro studies have recently reported the mechanisms of GL pharmacokinetics and GL-induced pseudoaldosteronism. We therefore attempted to use the method of mechanism-based modeling to make the risk control process more accurate and reasonable. Moreover, according to the opinions of the Scientific Committee on Food (SCF) [6] and the Dutch Nutrition Information Bureau (DNIB) [7], the upper safe limits for regular ingestion of GL are 100 mg/day and 200 mg/day respectively. However, because human toxicity studies remain incomplete, these upper limits may actually be too high to ensure sufficient protection to some subgroups. Consequently, modeling and simulation of scenarios difficult to undertake in clinical studies are needed to provide more accurate predictions of the dose limit in various populations. In addition, since the kidney is the target tissue of GA in GL-induced pseudoaldosteronism, determination of kidney exposure to GA in such populations is important to predict the likelihood of adverse effects of GL.

Physiologically-based pharmacokinetic (PBPK) modeling is now an established technique for estimating the absorption, distribution, metabolism and excretion of a drug and, in combination with pharmacodynamic (PD) modeling, predicting its pharmacological and/or adverse effects. Ploeger and colleagues developed a PBPK/PD model of GL in rat and human [8], [9], [10] and successfully predicted GL and GA plasma concentrations and the urinary cortisol∶cortisone ratio in human. However, in the Ploeger model, the urinary cortisol∶cortisone ratio did not indicate whether an adverse event had actually occurred. We therefore recognized the need to include in the model the clinical indicators of pseudoaldosteronism (e.g. serum potassium and sodium levels) and the kidney exposure to GA in order to better predict the adverse effects of GL. Moreover, once the MR is over-stimulated, the renin-angiotensin-aldosterone system (RAAS) is inhibited as a compensatory effect meaning RAAS also plays an important role in changing the levels of body fluids and electrolytes when GL is ingested.

In the present study, we first developed a semi-PBPK model for GL and GA in rat and then, based on parameters in the rat model and species scaling, developed a human PBPK model in which kidney exposure to GA was further combined with a biological system-PD model to produce a PBPK/PD model capable of predicting the indicators of GL-induced pseudoaldosteronism in human.

## Materials and Methods

### Experimental studies

During the modeling process, optimization and validation were conducted step by step using in vivo experimental datasets largely based on the literature. Moreover, in order to ask the validation more robust, we found it necessary to have data relating to the PK of GA and its kidney distribution after p.o. administration of GL. Two separate studies as follows were first conducted to provide a validation dataset for the rat PBPK model.

#### Materials

GL and GA (purity >98%) were obtained from Chia-tai Tianqing Pharmaceutical Co, Nanjing, China. Diclofenac for use as internal standard (IS) was purchased from Nanjing Chemical Reagent Co Ltd., Nanjing, China. All other chemicals were of analytical grade and used as received. Ultrapure water was used in all studies.

Male Sprague-Dawley rats (weight 220–250 g) were obtained from B&K Universal Group Ltd., Shanghai, China. The rats were housed under a 12 h light/dark cycle with free access to commercial rat chow and water. All animals were fasted overnight with free access to water before an experiment. All animal experiments were approved by the Animal Ethics Committee of China Pharmaceutical University and performed under a license granted by the Jiangsu Science and Technology Office (China). All efforts were made to minimize suffering.

#### Pharmacokinetics of GA after p.o. administration of GL

GL was dissolved in ultrapure water and administered p.o. to 5 rats at 100 mg/kg. Blood samples (0.2 ml) were collected at the following time points: 0.5, 1, 2, 4, 6, 8, 12, 24 h after the dose. Samples were stored at −20°C pending analysis.

#### Kidney distribution of GA after p.o. administration of GL

A group of 12 rats received a p.o. dose of GL at 200 mg/kg. After 9, 16 and 22 h, 4 animals were sacrificed at each time point by femoral artery exsanguination and blood and kidneys harvested. Each tissue was homogenized with an equal volume of cold saline after which samples were stored at −20°C pending analysis.

#### Analysis of GA in rat plasma and kidney

GA concentrations in the above samples were analyzed according to a previously developed method [11]. 90 µl plasma or tissue homogenate was taken into an Eppendorf tube, and added to 10 ml diclofenac (internal standard, 1 µg/ml) and 200 ml acetonitrile. The tube was vortex mixed and then centrifuged at 13000×g for 10 min. The supernatant was transferred to another clean Eppendorf tube and centrifuged at 13000×g for 10 min. An aliquot (5 µl) of supernatant was injected directly onto the HPLC column. And the analysis was carried out by LC-MS using an LC-MS 2010 system (Shimadzu, Kyoto Prefecture, Japan) comprising 2 LC-20AD pumps, a CBM-20A system controller, a CTO-20A column oven set at 40°C, a SIL-20AC autosampler set to inject 5 µL and a single quadrupole mass spectrometer equipped with an electrospray ionization (ESI) interface operated in the negative ionization mode. Separation was carried out on an Agilent Microsorb 100–5 C18 column (150×2.0 mm) using gradient elution with 0.1% acetic acid-10 mM ammonium acetate in water as mobile phase A and acetonitrile as mobile phase B at a fl ow rate of 0.2 mL/min. The gradient (min, %B) was as follows: 0–0.5, 20; 0.5–2.5, 40; 2.5–6.5, 90; 6.5–10.0, 20. Elution in the period 2.5–7.0 min was delivered into the mass analyzer. Detection was by selected ion monitoring (SIM) of ions with 469.3 for GA and 294.1 for diclofenac (internal standard IS). Optimum MS parameters were: Capillary voltage − 1.5 kV; nebulizer nitrogen flow rate 1.5 L/min; drying gas temperature 280°C; block temperature 220°C.

### Model building workflow

**Figure 1:**
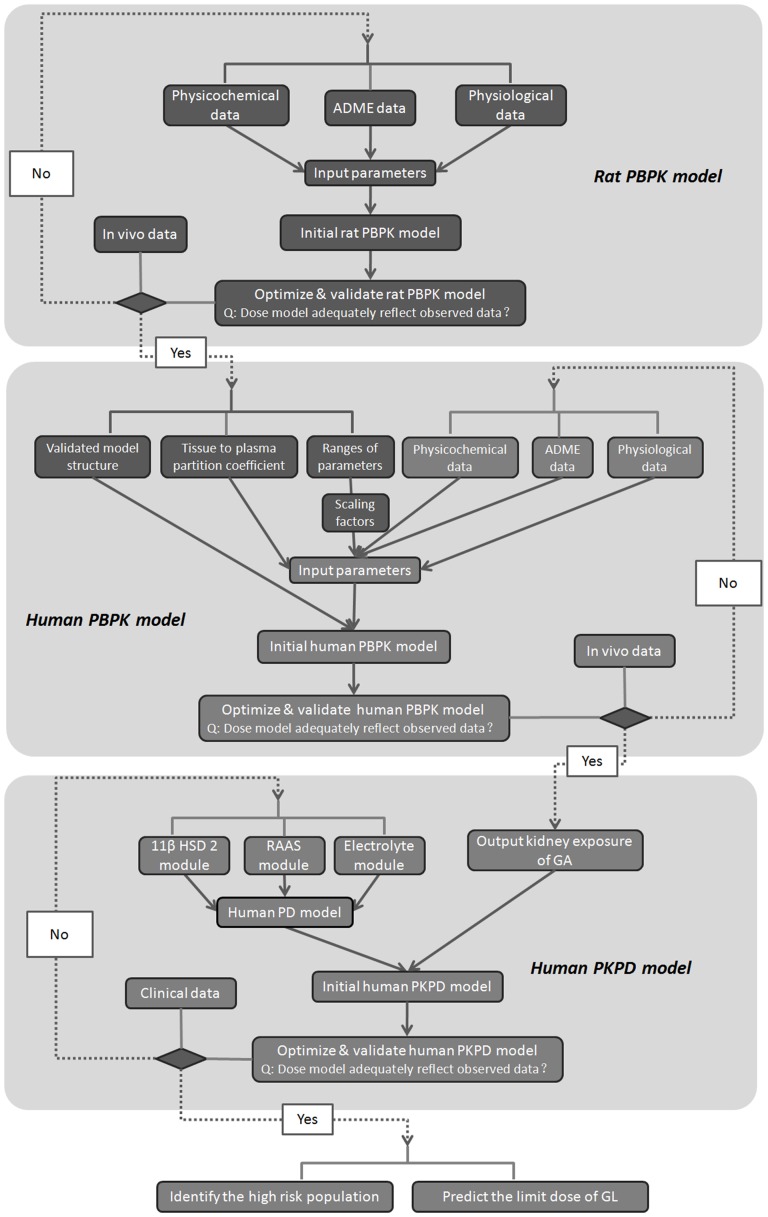
Work flow of PKPD modeling.

Following the proposed workflow shown in Figure 1, a PBPK/PD model for GL-induced pseudoaldosteronism was developed and used to predict the dose limit of GL producing pseudoaldosteronism in a virtual population. In brief, the rat PBPK model was first developed. Physicochemical parameters, absorption, distribution, metabolism and elimination (ADME) data, and physiological parameters were collected from the literature and used as initial input parameters of the model. After that, in vivo data sets were used: some to optimize, some to validate the model. If the model adequately reflected the observed data, the modeling process was continued; if not, the process would revert to the first step (see flowchart in figure 1). Based on the structure and parameters of rat PBPK model and also on scaling-induced extrapolation, we then developed the human PBPK model following similar steps as previously mentioned. After validation of the PBPK model, kidney exposure to GA in human was output and linked with the human PD model (consisting of three modules: 11β-HSD2 module, RAAS module and Electrolyte module). Finally, clinical indicators of pseudoaldosteronism could be predicted. The whole PBPK/PD model is developed step by step and validated during each step by observed data to prevent systematic errors.

### Description of the GL model

**Figure 2:**
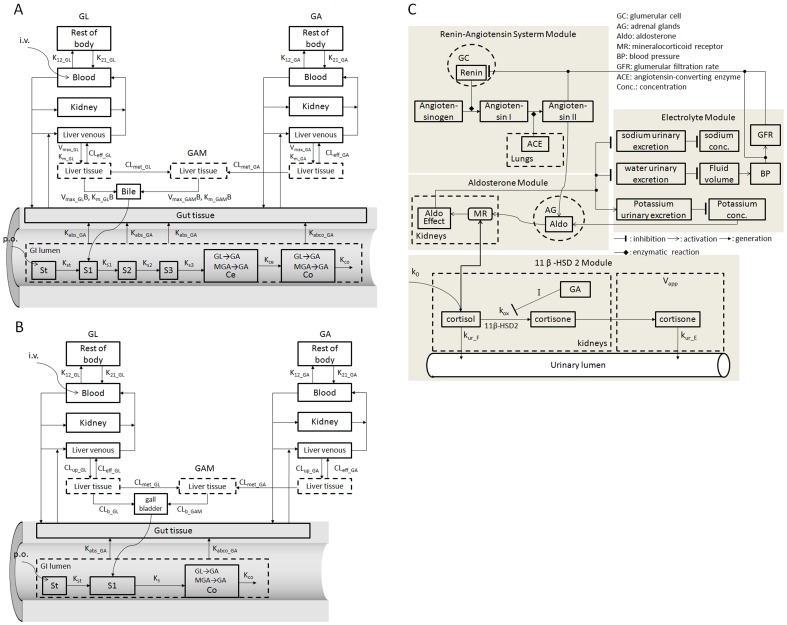
Model structure of the PKPD model. (A) Semi-PBPK model for the systemic kinetics of GL and GA in rat. (B) Semi-PBPK model for the systemic kinetics of GL and GA in human. (C)11β-HSD2 associated renin-angiotensin-aldosterone-electrolyte biological system PD model.

The model consists of four tissue compartments and one blood compartment as shown in Figure 2A. The abbreviation of the parameters used in the PBPK models are listed in Table 1. The kidney is the main target organ of GL-induced pseudoaldosteronism and has a high blood perfusion rate. The gut is isolated to describe the progress of absorption. Both of these tissues are assumed to be perfusion limited. As mentioned above, hepatic uptake and biliary excretion play important roles in GL elimination. GL has been shown to be actively transported into the liver by organic anion transport proteins (OATPs) [12] and subsequently excreted into the bile by multidrug resistance-associated protein 2 (MRP2) [13]. In order to model these processes, the liver was divided into a venous compartment and a tissue compartment and active transport of GL was described by a Michaelis–Menten-type equation. Besides biliary excretion, GL also undergoes passive diffusion from the liver into the plasma or is hydrolyzed to 3MGA by β-glucuronidase, both of which were assumed to be first order processes [14]. Then distribution in the remaining tissue was described by the parameters K_12_r_ and K_21_r_. Since urinary excretion of GL and its metabolites accounts for only a small proportion (<2.5%) of the total drug [15], [16], renal clearance was ignored for GL and its metabolites.

**Table 1:** Abbreviations of parameters of the physiologically based pharmacokinetic model for GL, GA and GAM.

Abbreviation	Parameter
A_abs_	Amount (µmol) of each compound absorbed from the gastrointestinal tract
A_x_	Amount (µmol) of each compound in blood (x = v), kidney (x = k), hepatic venous (x = vl), liver (x = l), gut tissue (x = g), bile (x = b), or remaining tissue (x = r)
CL_met_	Hepatic metabolic clearance (ml/h) of GL or GA
CL_x_	Hepatic sinusoidal uptake clearance (x = up) and biliary excretion clearance (x = b) (l/h) of GL or GA in human
C_x_	Concentration (µmol/ml) of each compound in blood (x = v), kidney (x = k), hepatic venous (x = vl), liver (x = l), gut tissue (x = g) or remaining tissue (x = r)
f_u_	The unbound fraction of GL or GA in plasma
f_ut_	The unbound fraction of GL, GA or GAM in tissue
K_12_r_	Clearance (ml/h) of each compound distributed from blood to remaining tissue
K_21_r_	Transit rate constant (h^−1^) of each compound from remaining tissue to blood
K_abs_x_	Absorption rate constant (h^−1^) of each compound in small intestine (x = si) or colon (x = co)
KH_x_	Hydrolysis rate constant (h^−1^) of GL or GAM in cecum (x = ce) or colon (x = co)
K_m_	Michaelis–Menten constant of each compound in hepatic sinusoidal uptake (µmol/ml)
K_x_	Gastrointestinal transit rate constant (h^−1^) in stomach (x = st), duodenum (x = sa), jejunum (x = sb), ileum (x = sc), cecum (x = ce) or colon (x = co) in rat model; or gastrointestinal transit rate constant (h^−1^) in stomach (x = st), small intestine (x = s) or colon (x = co) in human model.
PS_eff_	Hepatic sinusoidal efflux clearance of each compound (ml/h)
P_x_	Tissue to plasma partition coefficient in kidney (x = k) or gut (x = g)
Q_x_	Perfusion rate (ml/h) of kidney (x = k), hepatic artery (x = l) or portal vein (x = g)
V_max_	Maximum hepatic sinusoidal uptake rate of each compound (µmol/h)
V_max_B	Maximum biliary excretion rate of GL or GAM (µmol/h)

### Calibration of the GL model

Anatomical and physiological parameters taken from the literature [17] are listed in Table 2. Partition coefficients for each compartment [18] and the unbound fraction f_u_ of GL in plasma [19] are listed in Table 3. The unbound fraction of GL in tissue fu_t_ was estimated by equation (1) where fu_p_ is the unbound fraction in plasma, R is the average value of the tissue interstitial fluid-to-plasma ratio of albumin and lipoproteins (R = 0.5 for lean tissues and R = 0.15 for adipose tissue) [20], [21]:
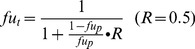
(1)


**Table 2:** Physiological parameters in a 250 g rat and 70 kg human.

Tissue volume and blood flow^c^				
	rat		human	
Compartment	Volume (ml)	Blood flow rate (ml/h)	Volume (l)	Blood flow rate (l/h)
Liver	10.3	866.6^a^	1.8	90.9^a^
Guts	13.75^b^	762	1.15	71.5
Kidneys	1.875	553.8	0.3	69
Hepatic artery		104.6		19.4
Portal vein		762		71.5
Liver venous	1.2		0.18	
Vascular	18.75		5	

Gastrointestinal properties^d^			
Compartment	Transit time in rat (h)		Transit time in human (h)
Stomach	1.33		0.4 for liquid; 1.8 for solid
Small intestine	Part 1	0.33	3.3
	Part 2	0.67	
	Part 3	2.5	
Large intestine	cecum	7	15
	colon	7	

a Sum of hepatic artery plus portal vein flows.

b Sum of small intestine and large intestine.

c from literature [17].

d from literature [8], [9].

**Table 3:** Biochemical parameters for the physiologically based model for GL, GA and GAM in rat.

Parameters	GL	GA	GAM	Source
K_12_r_	20	143	—	fitted
K_21_r_	0.46	1.6	—	fitted
f_u_	0.006	0.008	—	[19]; [49]
f_ut_	0.012	0.016	0.012	see text
Hematocrit	0.5	0.5	—	[19]
V_max_	9.0	9.0	—	fitted
K_m_	0.0014	0.0004	—	[22]; fitted
V_max_B	4.7	—	4.7	fitted
K_m_B	0.00037	—	0.00037	fitted
CL_met_	737	2666	—	fitted; see text
PS_eff_	26	411	—	[22]; see text
P_k_	0.14	0.15	—	[18]
P_g_	0.015	0.06	—	[18]; see text
K_abs,si_	—	0.47	—	[24]; fitted
K_abs,co_	—	0.58	—	[24]
KH_ce_	0.29	—	0.03	[23]; see text
KH_co_	0.11	—	0.03	[23]; see text

The initial values of V_max_ and K_m_ for hepatic uptake were taken as the values determined in an isolated rat liver perfusion study [22]. As V_max_ and K_m_ can change simultaneously to keep the ratio constant, we here fixed K_m_ to the value obtained from the literature on the assumption that drug affinity would not change much in the perfused rat liver. Then V_max_, K_12_r_ and K_21_r_ were optimized based on plasma concentration data and V_max_B, K_m_B and CL_met_ were optimized by curve fitting the accumulated biliary excretion data [15]. Since the oral bioavailability of GL is quite low (<4% in rat and cannot be detected in human) [5], [16], [23], [24], the absorption of GL after oral administration was ignored. The hydrolysis rate constant of GL (KH) in the large intestine was taken as the value determined by incubation of GL in gastrointestinal contents in vitro [23]. Other physiological parameters included in the gastrointestinal model were taken from the literature and are listed in Table 2 [17].

### Description of the GA model

As in the GL model, four tissue compartments and one blood compartment were modeled to describe GA disposition in rat (shown in Figure 2A). The kidney and gut compartments were as described for the GL model. As nearly 100% of the radioactivity in a dose of tritium-labeled GA was found to be excreted into bile (mainly as metabolites) [25], it is clear that the liver plays an important role in the disposition of GA. GA can competitively inhibit the hepatic uptake of GL [26] indicating that the two compounds share the same sinusoidal transport system. When transported into the liver, GA is metabolized mainly by UDP-glucuronosyltransferase and sulfotransferases in what are supposedly first order processes. The resulting phase II metabolites (GAM) are then excreted with bile into the intestinal tract and hydrolyzed to GA which then undergoes enterohepatic recycling.

### Calibration of the GA model

On the assumption that GA shares the same hepatic uptake transporter as GL, the V_max_ value which was considered to be related to the expression of the transporter was taken to be the same as that in the GL model leaving K_m_ to be optimized. The metabolic clearance of GA by the human liver (CL_in vivo, human_) was estimated based on its metabolism by human liver microsomes (HLM) in vitro [27] and further adjusted by the unbound fraction of drug in the incubation medium *f_u__*_inc_ using equations 2 and 3 where logD is the measure of lipophilicity of the compound [28].
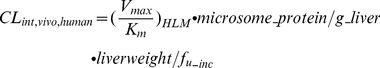
(2)

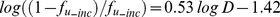
(3)


The metabolic clearance of GA by the rat liver (CL_int vivo, rat_) was then estimated by scaling on the basis if liverweight as given in equation 4:
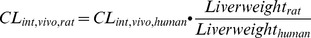
(4)


The absorption rate constants of GA in each intestinal segment were obtained by the in situ loop method provided by Ploeger et al [8], [24]. The efflux clearance of GA from the liver to the venous blood (CL_eff_) was assumed to be 15.6-fold greater than that of GL based on the fact that the passive permeability of GA in the gut lumen was estimated to be 15.6-fold greater than that of GL [24]. The data set of concentration-time profiles after i.v. administration of different doses of GA to rats with bile fistulas was used to optimize the parameters K_m_, K_12_r_ and K_21_r_ [29]. Like GL, the biliary excretion of GAM is via Mrp2 [5] for which V_max_B and K_m_B were assumed to be the same as those of GL and was validated using experimental data for: (1) The accumulated biliary excretion of 3MGA after i.v. administration of 3MGA at 5 mg/kg [5]; and (2) the accumulated biliary excretion of GAM after i.p. administration of GA at 25 mg/kg [25]. Then the colonic hydrolysis rate constant of GAM (KH_GAM_) was optimized according to the plasma concentration-time profiles of GA after i.v. administration of GA at 5.7 mg/kg in rats without bile fistulas [30]. All other parameters were obtained from the literature and are listed in Table 3.

Equations for the PBPK models of rat and human are listed in detail in the Appendix S1.

### Validation

The PBPK model in rat was further validated for the following scenarios: 10 mg/kg i.v., 25 mg/kg i.v. and 100 mg/kg i.v. GL [18], [19], [31]; 25 mg/kg i.p., 60 mg/kg i.v., and 5.7 mg/kg p.o. GA [18], [25], [30]; 10 mg/kg p.o. [30], 100 mg/kg p.o. and 200 mg/kg p.o. GL (experimental studies). The details are shown in the Results section.

### Development of human PBPK model for GL and GA: from rat model to human model

The structure of the model of GL and its metabolites in human is similar to that in rat shown in Figure 2B. As the absorptive properties of the compounds in the different regions of the gastrointestinal tract are unclear, we divided the gastrointestinal tract into only three parts viz the stomach, small intestine and large intestine. The physiological parameters in human are listed in Table 2. Moreover, in the human model, GL and GAM are stored in the gallbladder from which they undergo biliary excretion into the intestinal lumen after the ingestion of fat at meal times [9]. The rate constants for hydrolysis of GL and GAM in the gut lumen were calculated based on the literature [32] and K_12_r_ and CL_met_ in the human model were scaled from those in the rat model based on the cardiac output and liver weight respectively. Tissue to plasma partition coefficients for kidney (P_k_) and gut (P_g_) in human were assumed to be the same as those in rat.

The initial values of CL_up_ and CL_b_ in the human model were extrapolated from the corresponding clearances in the rat model using the allometric equations 5 and 6 where BW_rat_ and BW_human_ are the body weights of rat (250 g) and human (70 kg) respectively:
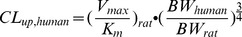
(5)

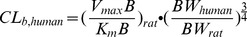
(6)


The absorption rate constant of GL during enterohepatic recycling in human was obtained from the literature [19]. CL_up_ and CL_b_ of GL were then optimized according to plasma concentration-time data for a single 120 mg i.v. dose of GL. The simulated profiles following other i.v. doses of 40 and 80 mg [16] were compared with the observed data to further validate the model.

CL_up_ and the absorption rate constant (K_abs_) of GA were optimized according to plasma concentration-time data for a single 130 mg p.o. dose [10]. The values were further validated for the following scenarios: Multiple p.o. administration of GA at 130 mg/day for 5 days [10]; a single p.o. administration of 225 mg GL; and a single p.o. administration of licorice equivalent to 225 mg GL [9]. For the licorice dose, the gut microbiota-mediated hydrolysis rate constant (KH) was assumed to be 4-fold greater since other components in licorice increase the extent of hydrolysis as shown in an in vitro study in rat [23].

### Development of the pharmacodynamic model in human

As mentioned previously, GA reversibly inhibits 11β-HSD 2 in the kidney and gives rise to pseudoaldosteronism. The scheme of the PD model for the 11β-HSD 2 associated renin-angiotensin-aldosterone electrolyte system is shown in Figure 2C. The 11β-HSD 2 module was first developed from the Ploeger model with some modification. The reversible inhibition of the conversion of cortisol into cortisone by GA can be described by the previously developed inhibitory Imax model [10],
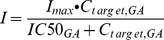
(7)where C is the concentration of GA in the kidney and IC50 is the concentration producing 50% of the maximum inhibition. The initial value of IC50 was assumed to be equal to the corresponding value found in rat kidney microsomes i.e. 0.32 µM [33]. Kidney exposure to cortisol was described by equation 8:
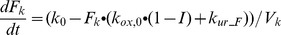
(8)where F_k_ is the kidney concentration of cortisol in human, k_0_ is the rate of cortisol formation (assumed to be constant), V_k_ is the kidney volume and k_ox_ and k_ur_F_ are the 11β-HSD 2 metabolic clearance and urinary clearance of cortisol, respectively. For simplicity, we assumed that the elimination of cortisol takes place by these two pathways only. We also assumed that the change in cortisone concentration in plasma was only due to its formation by 11β-HSD 2-mediated oxidation of cortisol and urinary excretion. Accordingly, the change in plasma concentration of cortisone was described by equation 9:
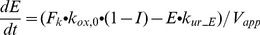
(9)where k_ur_E_ is the urinary clearance and V_app_ is the apparent distribution volume according to the plasma cortisone concentration. As the urinary cortisol∶cortisone ratio is the generally accepted biomarker of 11β-HSD 2 inhibition, the urinary content of cortisol and cortisone were calculated as follows:
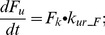
(10)

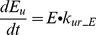
(11)and the 24 h urinary cortisol∶cortisone ratio (R) was described as: 
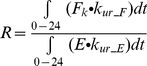
(12)


The parameters k_ur_F_ and k_ur_E_ were determined from the baseline 24 h urinary excretions of cortisol and cortisone [34] using the following equations:
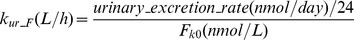
(13)

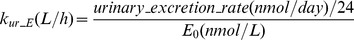
(14)

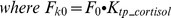
(15)


F_0_ and E_0_ are the baseline plasma concentrations of cortisol and cortisone respectively, F_k0_ is the baseline kidney concentration of cortisol and K_tp_cortisol_ is the kidney∶plasma concentration ratio of cortisol [35]. When GL is absent and equilibrium has been reached, equation 16 can be derived from equation 9:
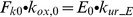
(16)and equation 17 from equation 8:



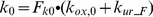
(17)


The IC50 and V_app_ value were optimized based on the 24 h cortisol∶cortisone ratio for 10 days after the first day of daily consumption of GA at 130 mg/day for five days.

Once 11β-HSD 2 is inhibited, the increased concentration of cortisol activates the MR and further induces sodium and water retention, hypokalemia, and hypertension. Ikeda et.al [36] developed a comprehensive network for overall regulation of body fluids which we used with some modification in the present study as the electrolyte module to predict the serum potassium and sodium levels. Moreover, several studies have reported that a significant suppression of the renin-angiotensin-aldosterone axis occurs after over-consumption of licorice and GA taken to be a compensatory response to the excessive activation of the MR by cortisol [34], [37]. Guillaud and Hannaert [38] developed a computational model of the renin-angiotensin system which can output the plasma renin, angiotensin I and angiotensin II levels but not aldosterone level, and successfully predicted the effect of renin inhibitor aliskiren. We then developed the mathematical relationship between aldosterone and angiotensin II given in equation 18 and further linked it with the Ikeda model shown in Figure 2C. Since angiotensin II and potassium are primary secretagogues modifying aldosterone release [39], we assumed that generation and secretion of aldosterone are mainly regulated by the two factors shown in equation 18 (i.e. the generation of Aldo stimulated by Angiotensin II and potassium in Figure 2C). Here K_gen_aldo_ and K_deg_aldo_ are the zero-order release rate constant and first-order elimination rate constant of aldosterone, S_max_ is the maximum stimulation, SC50 is the concentration producing 50% of maximum stimulation and r is the Hill coefficient. These parameters were obtained by curve fitting the experimental data [40], [41]. The RAAS is further regulated by the arterial pressure, venous pressure and glomerular filtration rate (GFR) which can be outputs of the Ikeda model [38].
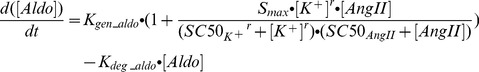
(18)


The body fluids model developed by Ikeda consists of 7 blocks, 5 of which were the main focus of the present study and are described briefly as follows: Block 1, the cardiovascular system, calibrates the relationship between blood volume and systemic arterial and venous pressure; Block 3, the extracellular space, calibrates the changes in water in the vascular and extracellular space; Block 4, the intracellular space and electrolytes, calibrates the potassium and sodium concentrations in extracellular fluids; Block 6, the kidney, calibrates the urinary excretion of water, sodium and potassium; and Block 7, the controller of renal function, calibrates GFR. Block 2 and 5 correspond to the predictions of the respiratory system and urinary anion excretion respectively which are not the main outputs in our model. The Ikeda model was described mathematically as a set of nonlinear differential and algebraic equations of more than 200 variables. Behavior of the model for various kinds of inputs simulated with a digital computer was in good agreement with a number of experimental results pertaining to body fluids and electrolytes [36]. The scheme of the renin-angiotensin-aldosterone-electrolyte network model modified on the basis of the original literature [36], [38] is shown in Figure 2C with equations given in detail in Appendix S2. The effect of cortisol on the MR was added to the aldosterone effect in the electrolyte module as indicated in equation 19 (i.e. the stimulation of MR by Aldo and cortisol in Figure 2C):
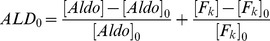
(19)where ALD_0_ is a normalized value presenting the effect of MR defined by Ikeda et al. and [Aldo]_0_ and [F_k_]_0_ are the baseline concentrations of aldosterone in plasma and cortisol in kidney respectively. ALD_0_ then affects the urinary excretion of potassium and sodium (YKU and YNU) in Block 6 of the Ikeda model. Finally the electrolyte module outputs the serum potassium and sodium levels.

### Simulation software

All simulations were performed using MATLAB (the MathWorks Inc., Natick, MA, USA). The PKPD model was constructed as a set of ordinary differential equation (ODEs), the integration of which was performed using the fourth order Runge-Kutta method. The accuracy of the prediction was graphically evaluated by superimposing the concentration–time profile observed *in vivo* to the simulated ones.

## Results

### PBPK modeling of GL and its metabolites in rat

**Figure 3:**
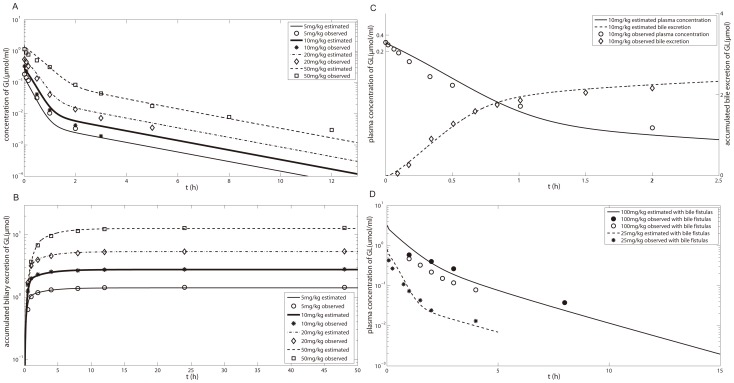
GL plasma concentration and accumulated biliary excretion after i.v. GL in rats with bile fistulas. Plasma concentration-time profiles of GL at (A) 5–50 mg/kg [15], (C) 10 mg/kg [13], (D) 25 and 100 mg/kg [18], [19], [31]; accumulated biliary excretion of GL at (B) 5–50 mg/kg [15] and (C) 10 mg/kg [13]. Experimental data (fitset and testset) are shown as symbols; the lines represent the prediction of the GL model.

In this study, a PBPK model was developed to predict the PK profiles of GL and GA after i.v. and p.o. administrations. Drug specific parameters used to develop the semi-PBPK model are listed in Table 3. Optimization of the parameters K_12_, K_21_, V_max_, V_max_B, K_m_B and CL_met_ in the GL model according to plasma concentration-time profiles and accumulated biliary excretion are shown in Figure 3A–B. It can be seen that the predicted lines give a good fit to the observed data. The optimized value of V_max_ (8.96 µmol/h) is quite close to that (10.1 µmol/h) calculated from isolated liver perfusion experiments, indicating that fixing K_m_ to the experimental value was a reasonable strategy. We further simulated different scenarios of i.v. administration of GL [18], [19], [31] and validated the model by comparing the simulations with data reported in the literature (Figure 3C–D). The results show that the simulated and reported profiles are comparable.

**Figure 4:**
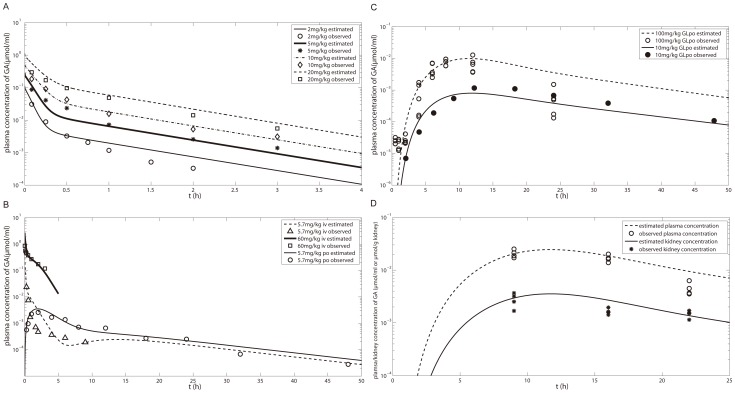
GA plasma and kidney concentration after i.v. GA and p.o. GA and GL in rats. (A) Plasma concentration-time profiles of GA after i.v. 2–20 mg/kg GA in rats with bile fistulas [29]; (B) i.v. 60 mg/kg GA in rats with bile fistulas [18], i.v. 5.7 mg/kg GA in rats without bile fistulas and p.o. 5.7 mg/kg GA in rats [30]; (C) p.o. 10 [30] and 100 (experimental studies) mg/kg GL; (D) plasma and kidney exposure of GA after p.o. 200 mg/kg GL (experimental studies). Experimental data (fitset and testset) are shown as symbols; the lines represent the forecast of the GA model.

**Figure 5:**
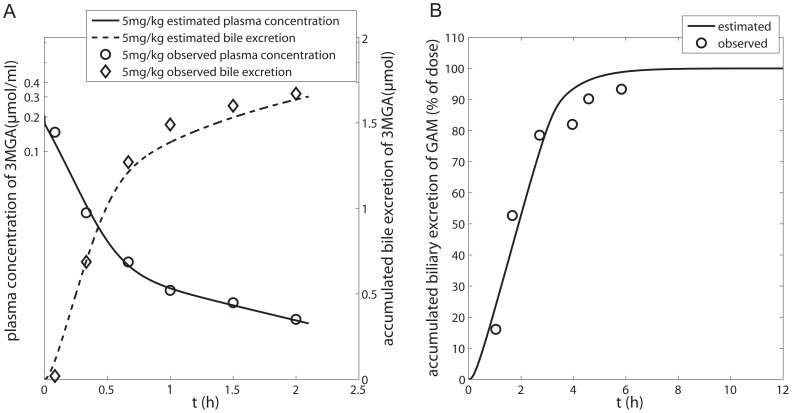
Plamsa concentration and accumulated biliary excretion of GAM. Left, plasma concentration and accumulated biliary excretion of 3MGA after i.v. 5 mg/kg 3MGA in rats [5]; Right, accumulated biliary excretion of GAM after i.p. 25 mg/kg GA in rats [25]. Experimental data are shown as symbols; the lines represent the predictions of the GA model.

Curve fitting of K_m_, k_12_ and k_21_ in the GA model to concentration-time profiles for i.v. administration of 2–20 mg/kg GA to rats with bile fistulas is shown in Figure 4A. Curve fitting of the 3MGA plasma concentration-time profile and subsequent simulation of its biliary excretion after i.v. administration of 3MGA at 5 mg/kg [5] and of GAM biliary excretion after i.p. administration of GA at 25 mg/kg [25] are shown in Figure 5. After optimization of KH_GAM_ by curve fitting of plasma concentration-time data for GA administration (i.v. 5.7 mg/kg [30]), the GA model was further validated for the following scenarios: 60 mg/kg i.v. [18] and 5.7 mg/kg p.o. GA; 10 mg/kg p.o. [30] and 100 mg/kg p.o. GL (experimental studies) shown in Figure 4B–C. Finally we simulated the kidney and plasma exposure to GA after p.o. administration of 200 mg/kg GL (experimental study) and compared the results with the observed data as shown in Figure 4D. The results show that the model predicts well the kidney exposure to GA which guarantees a reliable input into the PD model.

### PBPK modeling of GL and its metabolites in human

**Figure 6:**
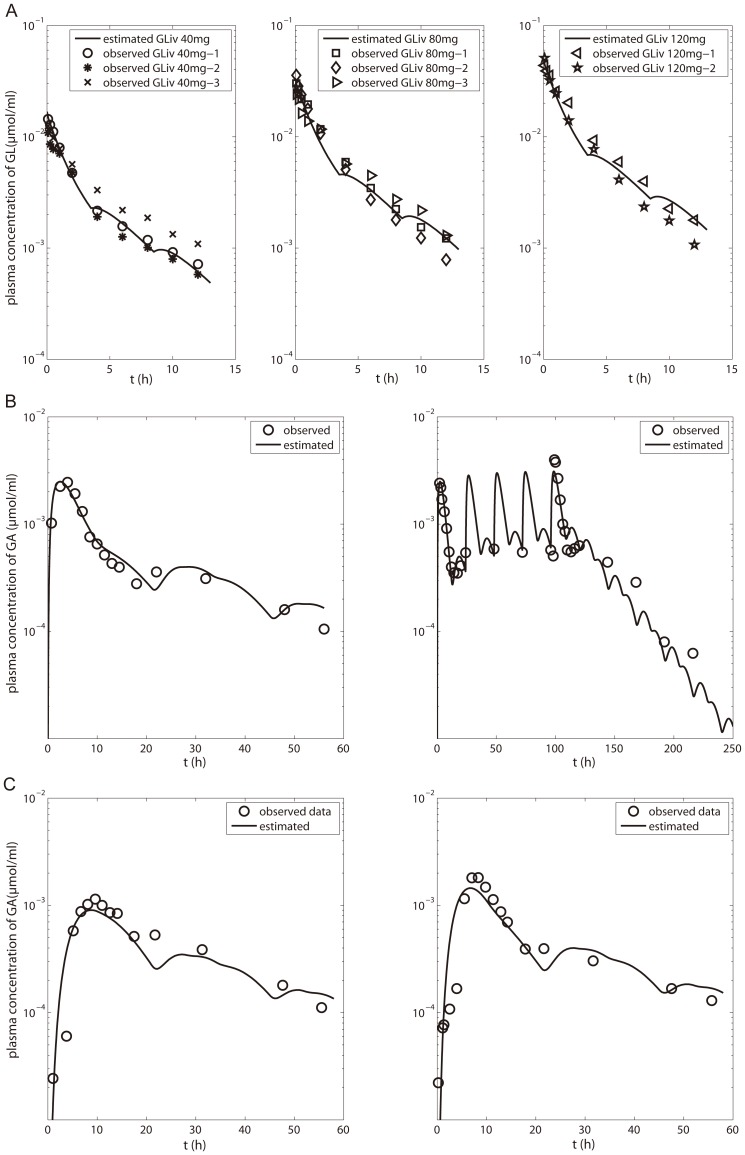
Plasma concentrations of GL and GA in human. (A) GL plasma concentration after i.v. 40–120 mg GL in human [16]; (B) GA plasma concentration after p.o. administration of 130 mg/kg GA at (B, left) single dose and (B, right) multiple dose [10]; GA plasma concentration after p.o. (C, left) 225 mg GL and (C, right) 150 g licorice containing 225 mg GL [9]. Experimental data (fitset and testset) are shown as symbols; the lines represent the predictions of the human PBPK model.

The estimated PK profiles following three i.v. doses of GL were comparable with literature data as shown in Figure 6A. In the GA model, the CL_up_ value and rate constants for absorption in the small intestine and colon were optimized based on the plasma concentration-time profile for p.o. administration of GA (Figure 6B). Simulation of the PK profiles of GA for the three scenarios of administration mentioned in the Methods (Figure 6B–C) illustrates that the model gives good predictions for the different scenarios. The drug specific parameters of the human PBPK model are listed in Table 4.

**Table 4:** Biochemical parameters of the physiologically based model for GL, GA and GAM in human.

Parameter	GL	GA	GAM	Source
K_12_r_	1241	9016	—	see text
K_21_r_	0.13	0.46	—	see text
f_u_	0.004	0.0008	—	[19]; [49]
f_ut_	0.008	0.0016	0.008	see text
Hematocrit	0.42	0.42	—	[19]
CL_up_	431	20000	—	see text
CL_b_	454.0	—	454	see text
CL_met_	50516	371546	—	see text
PS_eff_	1809	28179	—	see text
P_k_	0.14	0.15	—	see text
P_g_	0.015	0.06	—	see text
K_abs,si_	0.13	0.084	—	[19]; fitted
K_abs,co_	—	0.40	—	fitted
KH_co_	0.19	—	0.1	[32]

### PK/PD modeling for prediction of the adverse effects of GL in human

The human PK/PD model was constructed in two stages: the first to predict the urinary cortisol∶cortisone ratio (11β-HSD 2 module), the second to estimate the levels of serum potassium and sodium, plasma aldosterone etc. in the RAAS and electrolyte modules.

**Figure 7:**
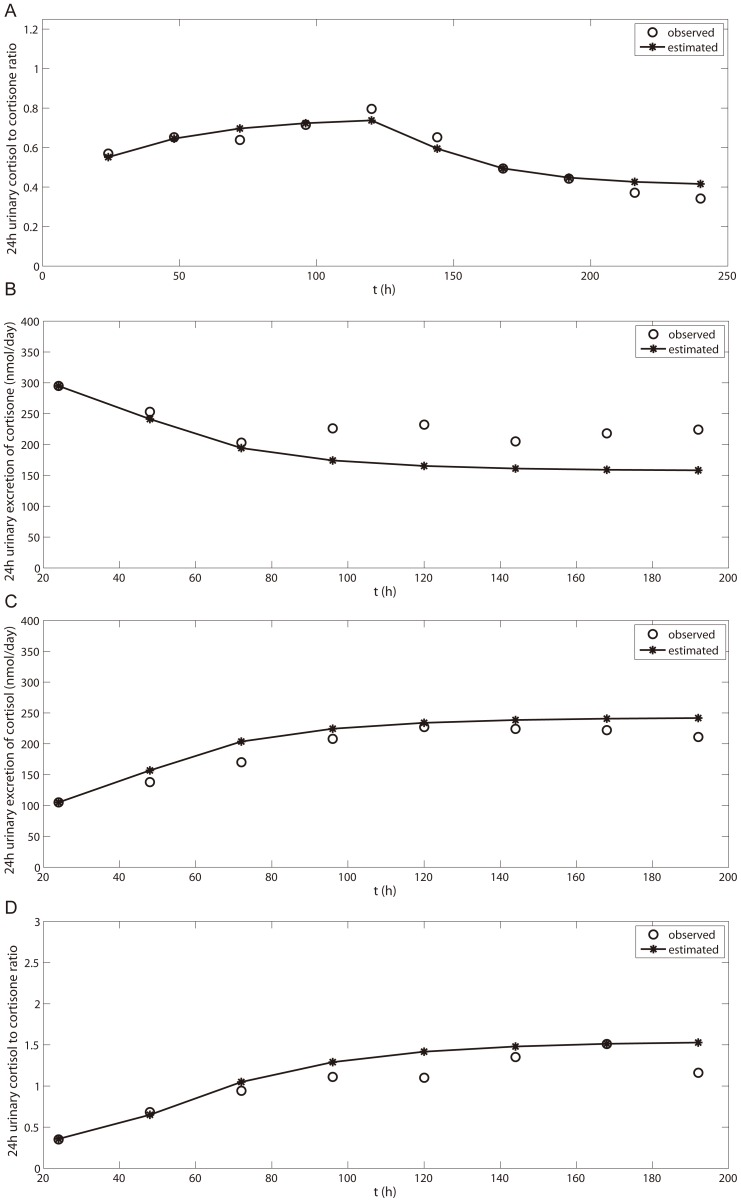
Time courses of urinary excretion of cortisol, cortisone and their ratio in different scenarios: (A) p.o. GA 130 mg/day for 5 days and withdrawn for another 5 days [10]; (B–D) p.o. GA 500 mg/day, 2 times/day for 7 days [34].

**Figure 8:**
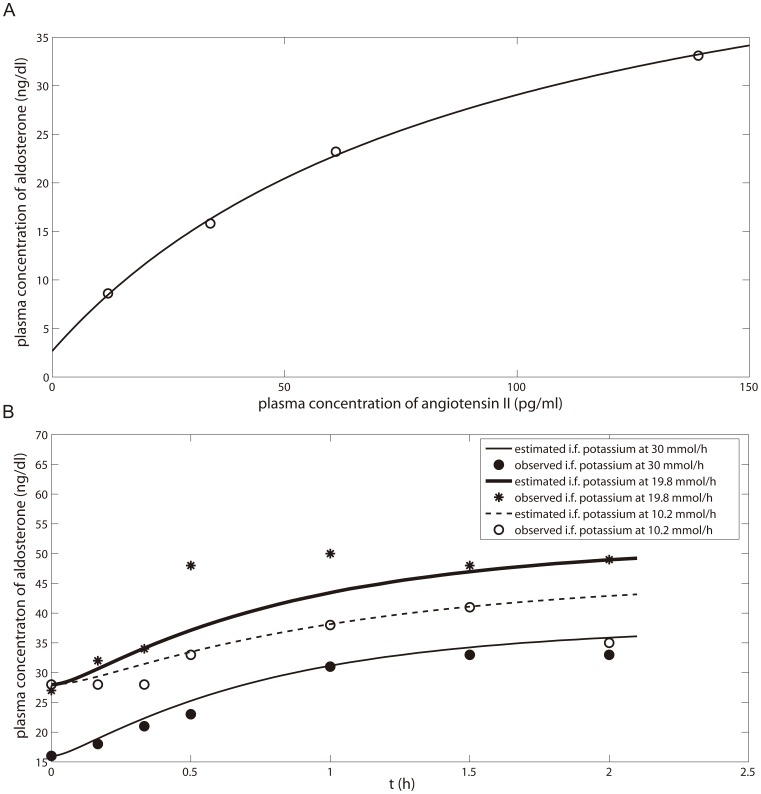
The effects of different levels of (A) angiotensin II [40] and (B) potassium [41] on aldosterone concentration. Experimental data (fitset) are shown as symbols; the lines represent the predictions of the human PBPK model. i.f. in B stands for intravenous infusion.

Figure 7A shows the results of optimization of the IC50 and V_app_ values based on the observed 24 h urinary cortisol∶cortisone ratio after p.o. administration of 130 mg/day GA for five days. The optimized parameters are listed in Table 5. Validation and simulation of the urinary cortisol∶cortisone ratio were further carried out for the following two scenarios: (1) Multiple p.o. administrations of 250 mg GA twice a day for 7 days (Figure 7D); and (2) multiple p.o. administrations of 170 mg GA three times a day for 2 days (Table 6). In the first scenario, we also simulated the 24 h urinary excretion of cortisol and cortisone and compared the results with the observed data (Figure 7B–C). It was found that the predicted and observed data were comparable indicating the resulting model was robust. Curve fitting the aldosterone model with the experimental data is shown in Figure 8A–B and the optimized parameters are listed in Table 5.

**Table 5:** Optimized parameters in the PD model.

Parameter	Optimized value
K_gen_aldo_ (ng/dl/h)	62.8
S_max_	92.63
SC50_K_^+^ (mmol/l)	4.577
SC50_AngII_ (pg/ml)	94.129
K_deg_aldo_ (h^−1^)	18.67
V_app_ (l)	5.2
IC50_GA_ (µmol/ml)	0.000234
Γ	9.97

**Table 6:** Estimated urinary cortisol∶cortisone ratio for p.o. administration of GA at 510 mg/day administered 3 times/day for 2 days compared with the observed value.

Dosage regimen	Time (day)	Observed value	Estimated value
GA p.o. 510 mg/day 3 times/day for 2 days	2	2.36 (0.69)^a^	2.0

a The observed value [50] is expressed as mean (SD). The baseline value before administration is 0.65 (0.06).

We then predicted the changes in the RAAS and serum electrolyte levels after p.o. administration of GA and licorice. The results shown in Tables 7 and 8 suggest that the RAAS was significantly inhibited presumably as a compensatory mechanism for over-activation of the MR as supported by experimental data from several studies. The predicted results after one and four weeks in the second scenario indicate that plasma levels of the indicators of GL-induced pseudoaldosteronism significantly declined in the first week consistent with the description in the literature. The predicted and observed values were all comparable showing that the indicators of pseudoaldosteronism are well predicted by the present PKPD model.

**Table 7:** Estimated biomarkers of pseudoaldosteronism for p.o. administration of GA at 500 mg/day 2 times/day for one week compared with the observed value.

	Base line	Observed value^a^	Predicted value
Aldosterone (ng/dl)	13.1 (2.7)	6.0 (3.5)	5.8
Sodium (mmol/l)	141 (0.5)	144 (0.6)	142
Potassium (mmol/l)	3.7 (0.09)	3.3 (0.04)	3.3

a The observed value [34] is expressed as mean (SE).

**Table 8:** Estimated biomarkers of pseudoaldosteronism for p.o. administration of GL at 0.7 g/day (9 subjects) or 1.4 g/day (5 subjects) for one to four weeks compared with observed values.

	Base line		Time after dose	Observed value^a^		Predicted value		
						0.7 g/day	1.4 g/day	mean
Aldosterone (ng/dl)	21.5		1 week	7.1 (1.9)		11.0	7.5	9.8
			4 weeks	8.6 (4.2)		8.9	5.4	7.7
Angiotensin II (pg/ml)	54.4		1 week	42.1 (14.5)		42.6	32.4	40.0
			4 weeks	40.8 (22.0)		46.6	37.8	43.4
Potassium (mmol/l)	**0.7 g/day**	4.1	4 weeks	**0.7 g/day**	**1.4 g/day**	3.7	3.3	
	**1.4 g/day**	4.0		3.7(0.4)	3.4(0.2)			

a The observed value [37] is expressed as mean (SD).

### Identification of the important factors relating to GL-induced pseudoaldosteronism

We further investigated the effects of changes in sinusoidal transport function, colonic transit time and activity of 11β-HSD 2, all of which have been reported to display high inter-individual variability, on the PK of GA and serum potassium level (chosen as the indicator of pseudoaldosteronism) after p.o. administration of 200 mg/kg GL in human.

**Figure 9:**
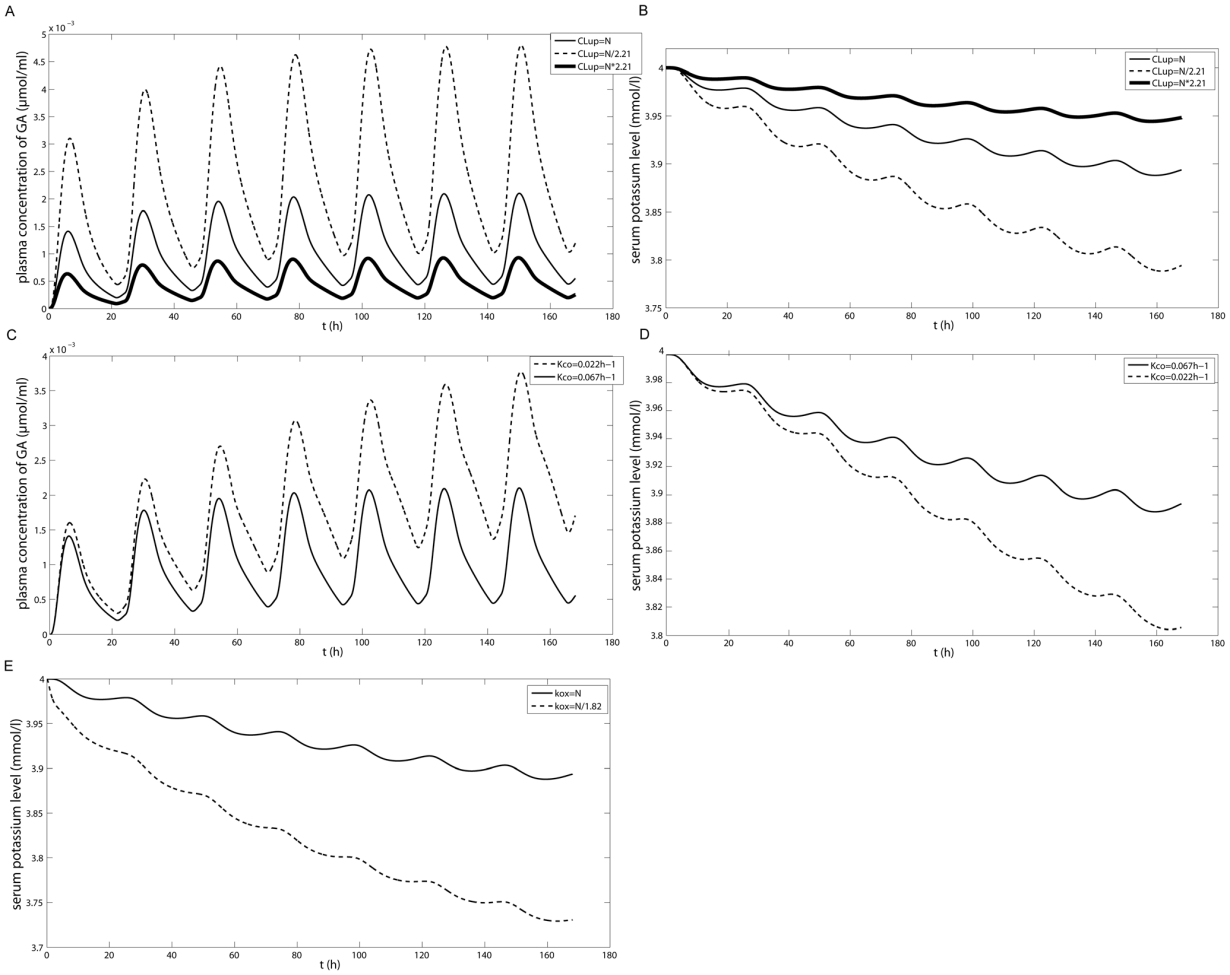
The simulated effect of three sensitivity factors on GA pharmacokinetics and potassium level. Sensitivity factors: (A, B) sinusoidal transport function, (C, D) colonic transit time and (E) activity of 11β-HSD 2. The causal effect: (A, C) GA plasma concentration and (B, D, E) serum potassium level. GL was administered p.o. at 200 mg/day for one week. N stands for the normal value of the corresponding parameter.

As mentioned above, hepatic clearance is the major elimination pathway of GA and, according to the parameters in the PBPK model of GA, hepatic sinusoidal uptake clearance (20000 l/h) is almost 1000 times greater than sinusoidal efflux clearance (28.18 l/h) meaning sinusoidal uptake rather than intrinsic hepatic metabolism is the determinant of hepatic disposition. Moreover, expression of the hepatic sinusoidal OATP transporters shows a large inter-individual variability suggesting that hepatic sinusoidal uptake function may contribute to the inter-individual variability in GL-induced adverse effects. CL_up_ is proportional to the V_max_ of the uptake process which is further supposedly proportional to the expression of the sinusoidal transporter. OATP1B1 and OATP1B3 are the major transporters mediating GL and GA sinusoidal uptake in human and regulation of their expression, according to previous reports, is coordinated within an individual [42]. Thus, in the present study, the change in expression of OATP1B1 was taken as a measure of the change in CL_up_. Details are as follows. According to a previous report [43], there is a 4.9-fold inter-individual variability in OATP1B1 mRNA expression level in the liver. Thus the median was set to the normal value N and two other values of CL_up_ (N/2.21 and 2.21*N) were chosen. The results (Figure 9A–B) show that a decrease in CL_up_ can significantly increase exposure to GA and subsequently decrease the serum potassium level, indicating that a decrease in sinusoidal transport function increases sensitivity to GL-induced adverse effects.

Since the adverse effect of GL is especially severe in elderly patients [44], [45] and since, according to previous reports, age is an important factor impacting colonic transit time and the activity of 11β-HSD 2 [46], [47], the effects of colonic transit time and activity of 11β-HSD 2 on the serum potassium level were further investigated. According to the reported values for colonic transit time and cortisol∶cortisone ratio in the elderly, we set K_co_ and k_ox,0_ to 0.0217 h^−1^ and 0.02 l/h respectively (the normal value of k_ox,0_ is about 1.8 times greater), predicted the plasma exposure to GA and potassium levels for one week and further compared them with those of using normal values of K_co_ and k_ox,0_. The results shown in Figure 9C–E suggest that a prolonged colonic transit time and a decrease in 11β-HSD 2 activity make subjects more susceptible to experiencing adverse effects and may explain why GL-induced pseudoaldosteronism mainly occurs in the elderly.

**Figure 10:**
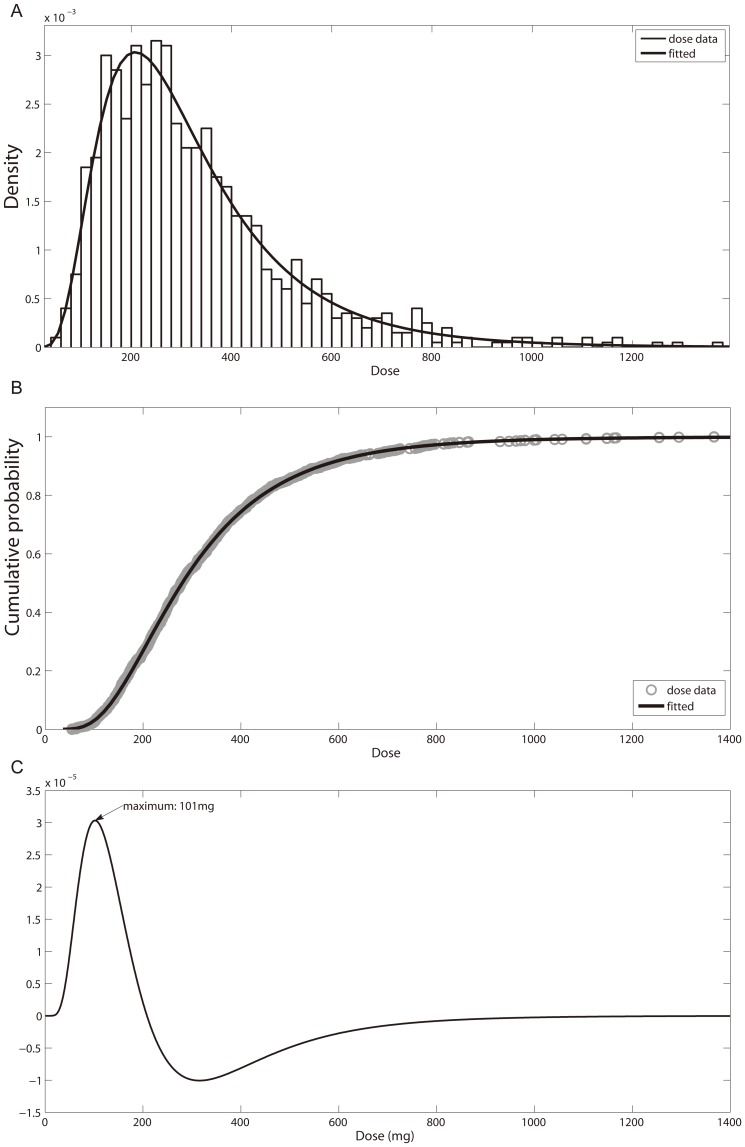
The simulated probability distribution of the individual dose limit in 1000 virtual elderly people. (A) The simulated dose data and fitted probability density by lognormal distribution; (B) The simulated dose data and fitted cumulative probability by log normal distribution; (C) The second derivative of the cumulative probability function and the critical value.

According to the literature mentioned above [43], [46], [47], the distribution of CL_up_ in the general population and K_co_ and k_ox,0_ in the elderly can be estimated and are listed in Table 9. Using the Monte Carlo method, we further simulated the dose limit for 1000 virtual elderly subjects according to the known distribution of the above three parameters in the elderly. Based on the normal range of serum potassium of 3.5–5.0 mmol/l, the median value (4.18 mmol/l) was chosen as the typical normal value of serum potassium. A series of doses were simulated for each subject and the individual dose limit leading to hypokalemia was determined as the dose giving a serum potassium level of 3.5 mmol/l on the 28^th^ day. The results are shown in Figure 10A. The distribution was well described by a logarithmic normal distribution and the distribution parameters μ and σ were found to be 5.6348 and 0.5449 respectively. Moreover, in the left part of the cumulative probability curve shown in Figure 10B, there is a critical value point of around 100 mg representing the highest rate of change in the cumulative probability. We further calculated it as the maximum value in the second derivative of the cumulative probability distribution function and obtained a value of 101 mg (Figure 10C). This indicates that the risk of adverse effects increases abruptly above 101 mg making this the critical dose limit of GL causing hypokalemia in the elderly with a probability of 3.07%. We further estimated the probability of hypokalemia in the elderly for the upper dose limit recommended by SCF (100 mg/day) as 3% and DNIB (200 mg/day) as 27%. The results show that, although the dose is only doubled, the probability is unexpectedly 9 times higher. It is suggested that the risk of the adverse effect at the DNIB recommended dose (200 mg/kg) is substantially greater probably due to high popularity of licorice containing foods in the Netherlands.

**Table 9:** Distribution of CL_up_, K_co_, and k_ox,0_ related physiological factors in simulation of the individual dose limit for 1000 subjects by the Monte Carlo method.

Physiological factor	Parameter	Number of samples	Estimation of the distribution	Sources
Expression of OATP1B1	CL_up_	31	Log normal distribution	[43]
Colonic transit time	K_co_	16	Bootstrapping	[46]
Plasma cortisol∶cortisone ratio	k_ox,0_	21	Truncated normal distribution^a^	[47]

a The boundary is according to the extreme value for 21 samples.

## Discussion

Consumption of GL in large amounts or for a long period of time leads to the adverse effects of pseudoaldosteronism which can be quite severe if ignored. In the present study, a semi-PBPKPD model was developed to predict the adverse effects of GL and facilitate its safe use both clinically and as a food additive.

Pseudoaldosteronism is caused by inhibition of 11β-HSD 2 by GA, the major metabolite of GL. Since the kidney is the target tissue for the adverse effects of GL, kidney exposure to GA is likely to be more important than systemic exposure and could be directly linked with the PD model. We made this prediction and validated it in the rat model and assumed that the plasma to kidney distribution in rat and human was the same. As a consequence, a semi-PBPK model was first developed in rat mainly to validate the model structure, clarify the mechanisms of action and provide reasonable parameters for the human model.

The present liver model was developed mainly on the basis of Ishida's isolated perfused liver model in which the parameters of GL hepatic disposition were calculated from experimental data and used as initial values in optimization. The structure of the PBPK model can be simplified or complicated according to the objective based on a reasonable mechanism. According to our objective of identifying a high risk population in which sinusoidal transport may be an important factor causing variability in adverse effects, the present model connected sinusoidal uptake, intrinsic hepatic metabolism and biliary excretion in a tandem pattern allowing analysis of the impact of sinusoidal transport on exposure to GL and GA. In contrast, in Ploeger's PBPK model, partial sinusoidal uptake and biliary excretion were combined into one process, making it difficult to further analyze the rate sensitive process in drug elimination.

Simple exposure-response PD models cannot explain the complicated mechanisms involved in such things as signal transduction and feedback loops making it difficult to predict clinical indicators far from the site of action. A mechanistic model can, however, include factors and processes involved in well-clarified mechanisms of action making it possible to simulate clinical reality, to predict more than one indicator at the same time and to establish the quantitative relationship between target-related and end point-related indicators. Thus, in order to achieve this objective, we developed a mechanism-based PD model. The PD model was further divided into three parts: the 11β-HSD 2 module (target related), the RAAS module (feedback related) and the electrolyte module (end point related). The 24 h urinary cortisol∶cortisone ratio is a generally accepted biomarker of the inhibiton of 11β-HSD 2 and sensitive to changes in the GA level in the kidney. In the present PD model, not only the ratio but also the urinary excretion of cortisol and cortisone could be predicted and further validated by comparison with experimental data. This made the further prediction of kidney exposure to cortisol more reasonable. In addition to the 24 h urinary cortisol∶cortisone ratio, serum electrolytes (potassium and sodium) and RAAS are also important indicators of GL-induced pseudoaldosteronism and are more indicative of the occurrence of adverse effects. In the present PD model, the plasma levels of angiotensin II, aldosterone, potassium and sodium were estimated and compared with the normal ranges of each indicator to predict the occurrence of adverse effects. Since there is no gold standard for GL-induced pseudoaldosteronism and since, of the various indicators of pseudoaldosteronism, serum potassium (hypokalemia) is an independent risk factor with the narrowest normal range (3.5–5 mmol/l) [48] and quite sensitive to the over-activation of MR [34], [37], we chose serum potassium as the main indicator in the simulation and prediction of the safe dose of GL.

After validation of the PKPD model, it was used to analyze the safe dose of GL. According to the SCF, a prudent dose of GL not exceeding 100 mg/day is recommended which is the LOAEL value in the most sensitive individuals. The simulation of the population mean in this scenario by the present model shows that 100 mg/day produces a less than 0.1 mmol/l decrease in the serum potassium level, which is considered safe for normal people. The screening results show that sinusoidal transport function, colonic transit time and the activity of 11β-HSD 2 are all important factors contributing to the inter-individual variability in experiencing adverse effects. In the present study, we considered the elderly as a high risk population and estimated the distribution of the individual dose limit based on data from recent studies.

In reality, describing an adverse effect should include three major factors: the dose limit, the specified population and the corresponding incidence of adverse effects. The present study provided the probability distribution of the individual dose limit in a virtual elderly population and calculated the critical value as the dose limit recommended by the present model. Interestingly, the critical value of 101 mg in our virtual elderly population was almost identical with the dose limit of 100 mg recommended by SCF. However, it must be pointed out that the SCF dose limit is an experimental value based on the clinically observed symptoms of pseudoaldosteronism while our critical value is based only on the serum potassium level. Also, the PKPD model provides the probability of increasing dose in a virtual population of 3.07% at the critical value which cannot be obtained from clinical observations of adverse effects due to ethical concerns. This is the most important advantage of modeling and simulation compared to clinical experiments. In further clinical trials, if new factors such as drug-drug interactions or the effects of polymorphism, or other metabolites of GL are discovered to be involved in producing adverse effects, they can be introduced into the model. In addition, the model can be used to simulate scenarios which may not be included in clinical trials.

In summary, we have developed a comprehensive PBPK/PD model based on PK data and pharmacology to predict the occurrence of GL-induced pseudoaldosteronism. In this model, not only the urinary cortisol∶cortisone ratio but also RAAS and serum potassium and sodium levels could be predicted. Furthermore, the present model simulated the probability distribution of the individual dose limit in a virtual population and provided the critical value of the dose limit to be recommended when GL is used as a food additive. This work would be useful to improve the safe use of GL as a food additive and drug.

## Supporting Information

Appendix S1 **Main equations of the PBPK model for GL and its metabolites in rat and human.**
(DOC)

Appendix S2 **Main equations in the PD model for pseudoaldosteronism.**
(DOC)
