# Supplementary material for: A Semi-Physiologically Based Pharmacokinetic Pharmacodynamic Model for Glycyrrhizin-Induced Pseudoaldosteronism and Prediction of the Dose Limit Causing Hypokalemia in a Virtual Elderly Population
Source: PLoS One. 2014 Dec 2;9(12):e114049. doi: 10.1371/journal.pone.0114049 (PMC4252094; doi:10.1371/journal.pone.0114049)
Supplement: Appendix S1 — Main equations of the PBPK model for GL and its metabolites in rat and human. (DOC) [file pone.0114049.s001.doc]

**Appendix S1**

**Main equations of the PBPK model for GL and its metabolites in rat**

Amount in blood:

Eq. (S1.1)

Amount in kidney:

Eq. (S1.2)

Amount in the remaining tissue:

Eq. (S1.3)

Amount in hepatic venous:

Eq. (S1.4)

Amount in liver:

GL: Eq. (S1.5)

GA: Eq. (S1.6)

GAM: Eq. (S1.7)

Amount in bile:

GL or GAM: Eq. (S1.8)

Amount in gut tissue:

$\frac{dA_{g}}{dt}=Q_{g}\bullet(C_{v}-\frac{C_{g}}{P_{g}})+\frac{dA_{abs}}{dt}$ Eq. (S1.9)

Amount in stomach:

Eq. (S1.10)

Amount in small intestine S1:

GL or GAM: Eq. (S1.11)

GA: Eq. (S1.12)

Amount in small intestine S2:

Eq. (S1.13)

Amount in small intestine S3:

Eq. (S1.14)

Amount in cecum:

GL or GAM: Eq. (S1.15)

GA: Eq. (S1.16)

Amount in colon:

GL or GAM: Eq. (S1.17)

GA: Eq. (S1.18)

Amount absorbed in the gut tissue:

Eq. (S1.19)

**Main equations of the PBPK model for GL and its metabolites in human**

Most equations in the human model were the same as those in the rat model except for the hepatic and gastrointestinal systems.

Amount in hepatic venous:

Eq. (S1.20)

Amount in liver:

GL: Eq. (S1.21)

GA: Eq. (S1.22)

GAM: Eq. (S1.23)

Amount in small intestine:

GL or GAM: Eq. (S1.24)

GA: Eq. (S1.25)

Amount in large intestine:

GL or GAM: Eq. (S1.26)

GA: Eq. (S1.27)
