# Supplementary material for: A Semi-Physiologically Based Pharmacokinetic Pharmacodynamic Model for Glycyrrhizin-Induced Pseudoaldosteronism and Prediction of the Dose Limit Causing Hypokalemia in a Virtual Elderly Population
Source: PLoS One. 2014 Dec 2;9(12):e114049. doi: 10.1371/journal.pone.0114049 (PMC4252094; doi:10.1371/journal.pone.0114049)
Supplement: Appendix S2 — Main equations in the PD model for pseudoaldosteronism. (DOC) [file pone.0114049.s002.doc]

**Appendix S2**

**Main equations in the PD model for pseudoaldosteronism**

Renin-Angiotensin System Module (according to previous report)

Cell renin content:

Eq. (S2.1)

Granular cell recruitment in one nephron:

Eq. (S2.2)

Total renin excretion:

Eq. (S2.3)

where F(X) is the regulation function of renin synthesis, secretion and GC recruitment. F(X) is regulated by blood pressure (arterial and venous), content of angiotensin II and GFR which can be calculated using the equations in the electrolyte module.

Systemic angiotensin I content (i.e. the generation of Angiotensin I from Angiotensinogen, see Figure 2C):

Eq. (S2.4)

Systemic angiotensin II content (i.e. the generation of Angiotensin II from Angiotensin I, see Figure 2C):

Eq. (S2.5)

Calculation of the variables and parameters in the above equations was the same as that in reference [38].

Electrolyte module (according to previously reported Ikeda model)

Table S2.1 Abbreviations for part of parameters or symbols in Ikeda model.

| General abbreviations | | | |
| --- | --- | --- | --- |
| ICF | intracellular fluid | | |
| GFR | glomerular filtration rate | | |
| Symbol | Normal value | | |
| DEN | | Proportional constant between QCO and VB | 1 |
| KR | | Parameter of right heart performance | 0.3 |
| PAS | | Systemic arterial pressure | 100 mmHg |
| PVS | | Systemic venous pressure | 3 mmHg |
| QCFR | | Capillary filtration rate | 0.002 l/min |
| QIN | | Drinking rate | 0.001 l/min |
| QIWL | | Rate of insensible water loss | 0.0005 l/min |
| QLF | | Rate of lymph flow | 0.02 l/min |
| QMWP | | Rate of metabolic water production | 0.0005 l/min |
| QVIN | | Rate of intravenous water input | 0 l/min |
| QWU | | Urine output | 0.001 l/min |
| RTOT | | Total resistance in systemic circulation | 20 mmHg.min/l |
| V_P_ | | Plasma volume | 2.2 l |
| VBRC | | Red blood cell volume | 1.8 l |
| YKIN | | Intake rate of potassium | 0.047 mEq/min |
| YKU | | Renal excretion rate of potassium | 0.047 mEq/min |
| YNIN | | Intake rate of sodium | 0.12 mEq/min |
| YNU | | Renal excretion rate of sodium | 0.12 mEq/min |
| ZKE | | ECF potassium content | 49.5 mEq |
| ZNE | | ECF sodium content | 1540 mEq |

The abbreviations for the parameters or symbols in the following equations are listed in table S2.1

Block 4 (i.e. the change in sodium and potassium conc. in Figure 2C):

Extracellular potassium content:

$\begin{matrix} \frac{d(ZKE)}{dt}=YKIN-YKU\text{+(transmenbrane }K\text{ shift exchanged with the H}^{\text{+}}\text{ transferred into }ICF) \\ -(\text{transfer into }ICF\text{ accompanying the secretion of insulin in the metabolism of glucose)} \end{matrix}$ Eq. (S2.6)

Extracellular sodium content:

$\frac{d(ZNE)}{dt}=YNIN-YNU+(\text{increase in Na exchanged with the H}^{\text{+}}\text{ transferred into }ICF)$ Eq. (S2.7)

Where YKU and YNU are regulated by aldosterone effects (see the path from ‘Aldo Effects’ to ‘sodium urinary excretion’ and ‘potassium urinary excretion’ in Figure 2C)

Block 3 (i.e. the change in fluid volume in Figure 2C):

Plasma volume:

$\frac{d(V_{p})}{dt}=V_{p}^{+}-V_{p}^{-}$ Eq. (S2.8)

$V_{p}^{+}=QIN+QVIN+QMWP+QLF$ Eq. (S2.9)

$V_{p}^{-}=QIWL+QWU+QCFR$ Eq. (S2.10)

Where YKU and YNU are regulated by aldosterone effects (see the path from ‘Aldo Effects’ to ‘water urinary excretion’ in Figure 2C)

Block 1 (i.e. the level of blood pressure in Figure 2C):

Arterial pressure:

Eq. (S2.11)

Venous pressure:

Eq. (S2.12)

Block 7 (i.e. the level of GFR in Figure 2C):

glomerular filtration rate:

$GFR=\left\{\begin{matrix} 0\text{ (PAS}<\text{40)} \\ 0.02PAS-0.8\text{ (40}\leq\text{PAS}<80) \\ -0.005(PAS-100)^{2}+1\text{ }(80\leq\text{PAS}<100) \\ 1\text{ (100}\leq\text{PAS)} \end{matrix}\right.$ Eq. (S2.13)

Calculation of the variables and parameters in the above equations is the same as in reference [36].
